# On a New and Simple Method for the Cure of Fistula

**Published:** 1852-03

**Authors:** H. B. Evans


*On a New and Simple Method for the Cure of Fistula.* By H. B. Evans, Esq., M. R. C. S., etc.—The frequent occurrence of fistula, and the often unfortunate and unsatisfactory results of an operation intended for its cure, induce me to make known to the profession, through the medium of the Lancet, a simple plan of treatment, which has proved eminently successful in two cases under my care.

In October, 1850, W. E——, box-maker, aged 42, applied to me with an abscess in the neighborhood of the rectum, pointing externally, which was opened, and gave exit to a large quantity of pus. This gradually degenerated into a deep fistulous tract along the rectum, and communicating with it at its extremity. For two months the usual remedies were adopted without success, and I then expressed my opinion that an operation must be resorted to. In this I was fully borne out by the opinion of an eminent hospital surgeon whom I called in. This the patient obstinately refused to submit to, and such refusal led to my adopting the mode of treatment I am abont to detail.

A blunt-pointed silver probe, five inches in length, (the sinus itself being four inches in depth), was inserted into the wound, having previously been dipped in dilute nitric acid (one part of acid to one part of water), and suffered to remain there a minute. That this had a strong cauterizing effect, I knew from the pain it occasioned. Thus far the result was desirable: but in consequence of the destruction of the silver probes by the acid, and the impossibility of using them more than three or four times, I had some copper ones made, and used them in the same manner, only thus substituting a nitrate of copper for a nitrate of silver, and I think with a better effect. Under this treatment I was pleased to see the depth of the sinus daily decrease by the gradual filling of it up with healthy granulations from the bottom. This was continued nearly every day for two months, February 22d, 1851, being the last occasion on which I thought it necessary to apply the nitrate of copper. The patient is at the present time perfectly sound.

In March, 1851, W. H——, aged thirty, applied to me with strumous disease of the testicle. Iodine and iron were given, which arrested the progress of the disease, and caused a corresponding improvement in his health. The outward form of the testicle was retained, but with an open sinus of an inch and a half in length in an oblique direction from the apex, and discharging a thin, white, glairy fluid, peculiar to fistula. The same treatment was pursued as in the former case, the sinus becoming entirely filled up, and the patient discharged at the commencement of the present month (September), without any external marks of previous disease, beyond a slight irregularity on the surface and a small cicatrix.

Thus by an easy method may the most strumous fistulæ be traced to their extremities, and a strong caustic power applied to the bottom of the wound, from whence it is so desirable granulations should arise.

A limited sphere of private practice enables me only to give these two cases; but I have no hesitation in saying, that if this system be approved of and practised by surgeons generally, they would have as much reason to be satisfied with it as myself and patients, and the use of the knife would become almost obsolete. When a silver and copper wire are introduced together, after having been dipped in the acid, the caustic effect is intense (likened by the patient to a red hot wire), and if allowed to remain too long, would destroy the tissues with which they were in contact. This, I apprehend, is the effect of the galvanic action set up by the contact of the copper and silver wire with the acid acting upon them.—*London Lancet.*

